# Conservation physiology and the quest for a ‘good’ Anthropocene

**DOI:** 10.1093/conphys/cox003

**Published:** 2017-02-15

**Authors:** Christine L. Madliger, Craig E. Franklin, Kevin R. Hultine, Mark van Kleunen, Robert J. Lennox, Oliver P. Love, Jodie L. Rummer, Steven J. Cooke

**Affiliations:** 1 Fish Ecology and Conservation Physiology Laboratory, Department of Biology and Institute of Environmental Science, Carleton University, 1125 Colonel By Drive, Ottawa, ON, CanadaK1S 5B6; 2 Department of Biological Sciences, University of Windsor, 401 Sunset Avenue, ON, CanadaN9B 3P4; 3 School of Biological Sciences, The University of Queensland, Brisbane, QLD4072, Australia; 4 Department of Research, Conservation and Collections, Desert Botanical Garden, 1201 North Galvin Parkway, Phoenix, AZ85008, USA; 5 Ecology, Department of Biology, University of Konstanz, Universitätsstrasse 10, D 78457 Konstanz, Germany; 6 ARC Centre for Excellence for Coral Reef Studies, James Cook University, Townsville, QLD4811, Australia

**Keywords:** Anthropocene, evidence-based conservation, pragmatism, public outreach, resilience

## Abstract

It has been proposed that we are now living in a new geological epoch known as the Anthropocene, which is specifically defined by the impacts that humans are having on the Earth's biological diversity and geology. Although the proposal of this term was borne out of an acknowledgement of the negative changes we are imparting on the globe (e.g. climate change, pollution, coastal erosion, species extinctions), there has recently been action amongst a variety of disciplines aimed at achieving a ‘good Anthropocene’ that strives to balance societal needs and the preservation of the natural world. Here, we outline ways that the discipline of conservation physiology can help to delineate a hopeful, progressive and productive path for conservation in the Anthropocene and, specifically, achieve that vision. We focus on four primary ways that conservation physiology can contribute, as follows: (i) building a proactive approach to conservation; (ii) encouraging a pragmatic perspective; (iii) establishing an appreciation for environmental resilience; and (iv) informing and engaging the public and political arenas. As a collection of passionate individuals combining theory, technological advances, public engagement and a dedication to achieving conservation success, conservation physiologists are poised to make meaningful contributions to the productive, motivational and positive way forward that is necessary to curb and reverse negative human impact on the environment.

## Teaser

The term ‘Anthropocene’ describes Earth's current geological period, which is characterized by human-induced changes to our atmosphere, geology and biodiversity. Although the narrative surrounding the fate of the world's species and ecosystems is often negative, many researchers are calling for strategies that can contribute to a ‘good Anthropocene’. We outline how the field of conservation physiology can infuse optimistic perspectives, pragmatic approaches and heightened public engagement into this pursuit.

An ounce of hope is worth a ton of despair. (George Monbiot, 2014)

## Introduction

The human presence on planet Earth is being felt more today than ever before as evidenced by massive levels of habitat alteration, pollution, environmental change and loss of biodiversity (Vitousek *et al*., 1997) that have knock-on effects on human society and well-being (e.g. Cardinale *et al*., 2012). Indeed, the rate of human-induced environmental change has been so profound that it is now widely accepted [but see Malm and Hornborg (2014) for dissenting view] that we are now in a new era distinct from the Holocene (Waters *et al*., 2016), which is called the Anthropocene (Crutzen, 2006; Steffen *et al*., 2007). The start of the Anthropocene is often attributed to the advent of the industrial revolution, or as the middle of the 20th century, and some scholars even propose that it began on the day of the Trinity nuclear explosion test in July of 1945 (Crutzen and Steffen, 2003; Steffen *et al*., 2011a; Lewis and Maslin, 2015; Waters *et al*., 2016).

Although the concept of the Anthropocene has inherent negative connotations (given the consequences of climate change, pollution and mass extinctions), some have refused to accept cataclysmic outcomes (i.e. the end of nature and humanity as we know it) and instead regard this as a rallying point; an opportunity to drive positive change in what has been called a ‘good Anthropocene’ (Szerszynski, 2012; Dalby, 2016). A good Anthropocene requires rethinking strategies for planetary stewardship (Steffen *et al*., 2011b) and identifying bright spots (see Bennett *et al*., 2016; Cinner *et al*., 2016) that can be leveraged, extended, embraced and applied in as many different ways as possible across the globe. Although a good Anthropocene means different things to different people, it is not a strictly preservationist perspective where good equals pristine. Given that humans are now a part of almost any ecosystem, elements of a good Anthropocene must recognize and incorporate the need for human development, infrastructure, services and use (Steffen *et al*., 2011b; Bai *et al*., 2016; Bennett *et al*., 2016; Dalby, 2016). As such, when faced with the question of what we want the legacy of the Anthropocene to be, many argue that it is only through a culture of hope, rather than one of ‘doom and gloom’ or despair, that we can rally individuals to act (Swaisgood and Sheppard, 2010; Hall, 2013).

Conservation physiology is a mission-oriented, multidisciplinary line of inquiry devoted to the application of physiological theory, knowledge, approaches and tools to the management of natural resources and conservation of biodiversity (Cooke *et al*., 2013). As this nascent research area comes into its own and is embraced by the broader scientific community (Lennox and Cooke, 2014), it is becoming apparent that the field has already generated a number of success stories (reviewed by Madliger *et al*., 2016) supported by a robust conceptual framework (see Coristine *et al*., 2014) that emphasizes connections between the science and its application. Not surprisingly, as a group of researchers and practitioners active in the realm of conservation physiology and committed to achieving a good Anthropocene, we have spent considerable time considering how to use our tools and approaches to achieve positive outcomes. To that end, here we outline how the discipline of conservation physiology can help to define what we want in a good Anthropocene and how to achieve that vision. For reference, we have viewed ‘defining a good Anthropocene’ as delineating a productive, motivational and positive way forward in our attempt to curb and reverse negative human impact on the natural world. It is our hope that this proposed vision will help to shape the continued development of the discipline (both in terms of discovery and training), direct the content of the journal *Conservation Physiology* and, most importantly, help to achieve the good Anthropocene that we all desire.

## Conservation physiology's role in defining and achieving a good Anthropocene

Here, we summarize four major ways in which conservation physiology can promote a good Anthropocene, as follows: (i) building a proactive approach to conservation; (ii) taking on a pragmatic perspective; (iii) establishing an appreciation for environmental resilience; and (iv) engaging in the public and political arenas. We consider these approaches to be inherent properties and/or goals of conservation physiology and therefore we envision that the discipline can contribute substantially to defining a good Anthropocene by making contributions to some or all of these objectives [e.g. Box 1: studies of impacts of dredging on clownfish (*Amphiprion percula*) and captive breeding programmes for the black rhinoceros (*Diceros bicornis*)]. Importantly, many of the specific examples included herein are relevant to multiple goals, which we view as a testament to conservation physiology's broad applicability, diversity and its focus on a well-documented, experimentally derived evidence base.

Box 1: **Conservation physiology leads by example to promote a good Anthropocene. Here, we present two case studies that highlight how conservation physiology is able to contribute a proactive approach, take a pragmatic perspective, appreciate resilience and engage the public to address conservation challenges. Within each component of the case studies, we have indicated the specific task that conservation physiology has accomplished. It should be noted that each of the case studies was able to contribute to all four goals, but that this will not necessarily be the case in every scenario; research that can contribute to even one goal can still have immense impact on promoting a positive path in the Anthropocene.**

**Case Study 1: dredging near the Great Barrier Reef—impacts on native clownfish (*****Amphiprion percula***). With the expansion of a shipping port in Abbot Point in Australia, sediment and dredge spoil are threatening the associated coral reef ecosystem (Australian Coral Reef Society, 2015). Physiological work investigating growth, development, respiration and the microbiomes of clownfish larvae (Hess *et al*., 2015) is supporting management recommendations for restoration, guiding strategies to minimize impacts on coral and reef fish spawning (J. Rummer, personal communication) and engaging the public (e.g. Brisbane Times, 2015; Nature World News, 2015) and not-for-profit organizations (J. Rummer, personal communication).

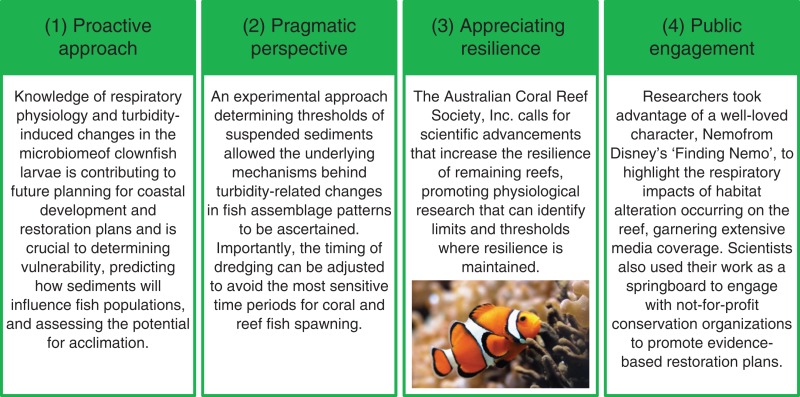

**Case Study 2: black rhinoceros (*****Diceros bicornis*****) captive breeding and conservation.** Black rhinos are critically endangered and have been the focus of captive breeding programmes around the world. Monitoring of reproductive hormones in faeces of wild and captive individuals has contributed to markedly improved breeding success by providing detailed information on puberty, cycling, optimal insemination times and the diagnosis and monitoring of pregnancy (Edwards *et al*., 2015a, b; Santymire *et al*., 2011). Importantly, the success of the programmes has been shared with visitors and online media outlets (BBC News, 2013; University of Liverpool, 2014; Conservation Careers, 2016).

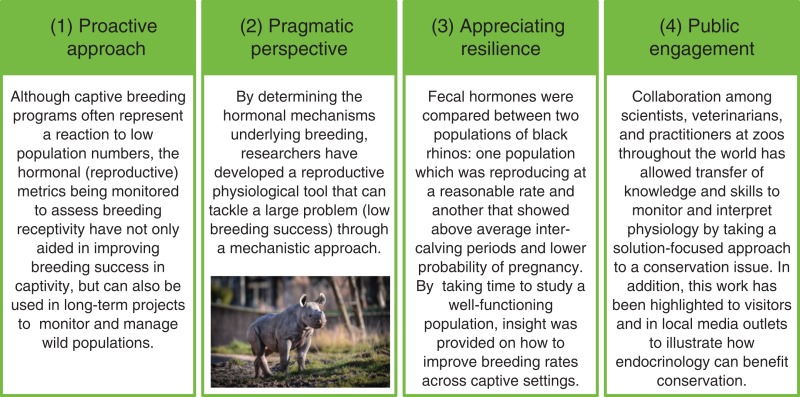

### A proactive, rather than reactive, approach to conservation

Although conservation biology is often viewed as a ‘crisis discipline’ (Meine *et al*., 2006), the value of proactive efforts for limiting biodiversity loss are increasingly accepted as highly valuable approaches along a prioritization continuum (Tabarelli and Gascon, 2005; Brooks *et al*., 2006; Bush *et al*., 2014; Drechsler *et al*., 2011). In particular, placing effort on preventing or reversing changes before they become catastrophic represents an inherently optimistic approach that emphasizes what can still be accomplished within a given situation (i.e. how to achieve success), rather than what must be accomplished to correct a failure. From a purely practical perspective, proactive approaches are estimated to be less economically costly compared with their reactive counterparts (Keller *et al*., 2007; Drechsler *et al*., 2011), which enables more projects to be accomplished within an inevitably limited budget. Importantly, conservation triage that begins early can lean on physiology, not only to be better prepared to tackle the consequences of further change, but also, potentially, to accomplish more than could happen by relying solely on reactive approaches that often necessitate rushed decisions based on limited evidence. As conservation physiology already takes a proactive approach in many capacities, the discipline can continue to do so in the following ways.

#### Providing baselines, quantitative definitions of ecosystem integrity and management goals

By providing measureable traits, conservation physiology allows limits (i.e. thresholds) to be quantified where conditions may destabilize populations (e.g. through reductions in fitness), species, communities or ecosystems, thus allowing for proactive management within quantitative ranges. When thresholds are surpassed, physiological knowledge can also provide insight on how to reverse these changes most effectively. For example, measurement of fatty acid profiles, antioxidant capacity, haematocrit and total serum protein concentrations in southern stingrays (*Dasyatis americana*) inhabiting areas exposed to ecotourism (direct interaction from feeding and touching) allowed management strategies to be formulated regarding boat numbers, visitor numbers and the composition of artificial foods before detrimental impacts on life expectancy and population size occurred (Semeniuk and Rothley, 2008; Semeniuk *et al*., 2007, 2009, 2010). In addition, when targets are set for management actions, practitioners will be able to assess when they have accomplished a goal, and continuously verify that their systems are functioning.

#### Producing well-populated models for use as decision-support tools

Understanding variations in physiological traits can help to predict patterns in ecological phenomena, such as movement (Breau *et al*., 2011), settlement/habitat selection (Blouin-Demers and Weatherhead, 2001), social behaviour (Koolhaas *et al*., 1999), reproductive timing and phenology (Wilczek *et al*., 2010) and foraging locations (Whitlock *et al*., 2015). In particular, models involving physiology are expected to be extremely relevant to predicting the spread of invasive alien species (e.g. Australian *Acacia* and *Eucalyptus* tree species: Higgins and Richardson, 2014; cane toad *Rhinella marina*: Kolbe *et al*., 2010; Seebacher and Franklin, 2011; Winwood-Smith *et al*., 2015), disease dynamics (Altizer *et al*., 2013; Ceccato *et al*., 2016), responses to global warming (Kearney and Porter, 2009) and delineation of source vs. sink populations (e.g. Whitlock *et al*., 2015). The incorporation of physiological traits into modelling should provide unprecedented forecasts of how organisms make decisions, ultimately allowing practitioners to manage habitats spatially and temporally at potentially finer scales and with lower labour and monetary requirements.

#### Imparting predictive monitoring capacity

Monitoring physiological traits that respond to environmental variation can provide insight into how organisms translate extrinsic environmental information into internal responses that ultimately impact performance (Cooke *et al*., 2013). For example, physiological stress responses have been shown to predict the vitality of white sturgeon (*Acipenser transmontanus*) following fisheries stressors and have been linked to relatively simple and inexpensive behavioural indices that could be used in a field setting (McLean *et al*., 2016). In addition, Farrell *et al*. (2008) showed that models of aerobic scope can be used to predict Pacific salmon (*Oncorhynchus* spp.) migration success, providing an opportunity for predictive capacity for this species, given the growing concern of climate change. Conservation-focused monitoring that uses physiological biomarkers therefore has the potential to detect deviations sensitively so that mitigation can begin before individuals or systems have lost the capacity to recover.

### A pragmatic perspective

In the face of constraints on funding, labour and other logistics, it is inevitable that trade-offs will occur when deciding where, when and how much to conserve (Wilson *et al*., 2007; Kareiva and Marvier, 2012). Although these constraints may appear largely negative, it is possible to approach such decisions from a positive perspective that strives for efficiency, cost-effectiveness and prioritization of threats (Wilson *et al*., 2007). For example, we may be required to use approaches and set goals that are more likely to be accomplishable rather than overwhelming, which can have a motivational effect. Furthermore, incremental successes can foster further progress by providing worked examples, supporting individuals who can pass skills and knowledge across a community, and compiling encouraging stories that inspire a new generation of volunteers and professionals. Indeed, messages of ‘hope’ have been deemed necessary to attract youth to professional careers in conservation (Swaisgood and Sheppard, 2010) and to engage the public more broadly (Chapman *et al*., 2015). At its core, conservation physiology is a pragmatic discipline which, based on the successes it is currently accumulating (reviewed by Madliger *et al*., 2016), can help to define how a practical perspective can be worthwhile by allowing us to accomplish the following tasks.

#### Possess a well-equipped and validated toolbox

Given that conservation physiology is a discipline characterized by the use of diverse field and laboratory technologies, it continues to promote novel methodologies to document, predict and mitigate environmental change. Much of this technological advancement stems from the field's ability both to integrate across a broad range of disciplines (molecular genetics and genomics, human physiology and athletic science, veterinary science, medicine and many others) and to repurpose technologies developed within other fields for use in wildlife and other organisms (Cooke *et al*., 2013). Overall, this imparts conservation physiology with a rapidly evolving toolbox capable of drawing on a variety of cutting-edge disciplines that are refining tools and techniques simultaneously.

#### Characterize and appreciate how organisms function in specific environments

By characterizing physiological traits over gradients or discrete classes of environmental quality, conservation physiology can determine tolerances where environmental change may influence reproduction, photosynthesis, energetics, immune function and other physiological processes (Wikelski and Cooke, 2006). Beyond helping to determine these types of thresholds in the context of stemming environmental change, this type of approach can also help in selecting candidate taxa for restoration of degraded ecosystems (Cooke and Suski, 2008). For example, Pywell *et al*. (2003) completed a meta-analysis that indicated that the performance of plant species in restored vegetation communities in Great Britain was related to a variety of physiological and morphological traits, allowing managers to understand how different species might potentially respond to restoration. Likewise, by examining physiological traits related to growth and water uptake, Walker *et al*. (2004) determined that ectomychorrhizal inoculation (by the fungus *Pisolithus tinctorius*) promoted establishment of sweet birch (*Betula lenta*) on surface mine spoil without the need for intensive application of chemical fertilizer. Beyond restoration, determining physiological performance can also improve the success of captive breeding programmes (Wingfield *et al*., 1997; Box 1), aid in the design of reintroduction plans for native species (Tarszisz *et al*., 2014) and inform eradication strategies for invasive organisms (Gardener *et al*., 2010).

#### Tackle large problems using a mechanistic approach

Given that conservation physiology focuses on the mechanisms underlying organismal response to environmental change, it is inherently suited to identifying cause-and-effect relationships (Carey, 2005; Cooke *et al*., 2012; Seebacher and Franklin, 2012). Most importantly, knowledge of underlying mechanisms can be an advantage when designing on-the-ground conservation solutions. For example, an understanding of sensory and reproductive physiology of invasive alien species can assist in capture, transfer or inhibition of reproduction for the purpose of controlling spread (e.g. sea lamprey *Petromyzon marinus* in the Laurentian Great Lakes: Youson, 2003; Wagner *et al*., 2006). Ideally, fine-scale physiological data will allow for these types of scenarios without interfering with related, endemic species in an ecosystem. Alternatively, an understanding of physiological function can also aid in designing infrastructure that does not interfere with wildlife, such as lighting, aircraft and structures in open landscapes (D'Angelo *et al*., 2005; Navara and Nelson, 2007; Martin and Shaw, 2010; Blackwell and Fernandez-Juricic, 2013). Overall, we anticipate that large-scale conservation challenges, such as the spread of infectious disease, captive breeding and reintroduction programmes, control of invasive alien species (see Lennox *et al*., 2015) and interaction of wildlife with human structures, will all benefit from considering physiological mechanisms, and success stories are already accumulating (Madliger *et al*., 2016).

#### Accomplish evidence-based conservation

Currently, there is a push toward evidence-based approaches in all sub-disciplines of conservation to improve knowledge transfer, eliminate inefficient trial-and-error strategies and increase success of management initiatives (Sutherland *et al*., 2004; Bainbridge, 2014; Legge, 2015; Walsh *et al*., 2015). Approaches that do the most with what is already available are essential to promoting a good Anthropocene; we will maintain a positive outlook on what can be accomplished only if we continue to forge ahead, record our successes (as well as failures) and disseminate successes to relevant stakeholders and the public. As Sutherland *et al*. (2004) outlined, an evidence-based approach to conservation should not only be more effective, it should also allow researchers and organizations to garner greater funding to support their work. Conservation physiology embodies the ideals of evidence-based conservation because it is constantly accumulating knowledge that can be evaluated for patterns, emerging themes and applicability to alternative scenarios. Even with the formal naming of the discipline 10 years ago, Wikelski & Cooke (2006) stated, ‘a conservation-physiology database needs to be established that enables conservation managers to quickly identify the most appropriate solution to conservation problems’. Revisiting this as a goal for the field is one way in which conservation physiology could be a torchbearer for the evidence-based conservation that must form the foundation of a good Anthropocene movement. We stress that the construction of the database will be of great importance to allow individuals to access the information they desire (i.e. balancing the amount of information contained with the ease with which it can be extracted). In addition to this, conservation physiologists can promote evidence-based conservation by placing effort on publishing not only significant but also null results.

### An appreciation for the resilience of many species in the face of environmental change

Although many organisms are negatively affected by environmental degradation and climate change, there are many examples of acclimation, adaptation, range expansion and resilience (Kareiva and Marvier, 2012; Seebacher *et al*., 2015; van Kleunen *et al*., 2015). Specifically, conservation physiology gives us the opportunity to observe, document and appreciate this resiliency and to interpret why some organisms or populations are better able to overcome or prosper in the face of change compared with others (e.g. by altering physiology and behaviour to cope with challenges without detrimental consequences to persistence). For example, a meta-analysis by Seebacher *et al*. (2015) indicated that plasticity in physiological rates (metabolic rate, heart rate, enzyme activity and locomotor performance) increases the resilience of ectothermic animals to climate change. By having this type of physiological information, we can start to delineate where best to focus time, energy and resources (i.e. offer help where it is most needed). Overall, this illustrates how a discipline that can acknowledge all the consequences of environmental change (negative, neutral and positive) could aid in the development of more efficient conservation strategies.

### An informed and engaged public and political realm

Conservation physiology is poised to play a leading role in narrowing the research–implementation gap and establishing connections between the public and science because it represents a marrying of technology and passionate individuals. Importantly, being a relatively nascent field, it comprises eager researchers who are pressing for growth, thinking critically about the potential for and future of the field and sharing ideas about how to achieve those goals. When it comes to engaging politicians and the public, we urge researchers to place emphasis not simply on the quantity of scientific information that can be generated, but also on how we can successfully communicate findings to those outside the scientific community (Box 2). Within this framework, conservation physiologists have the capacity to be both scientists and advocates via the following methods.

Box 2: **Opportunities for conservation physiologists to increase participation and effectiveness in science communication. We encourage all researchers in the field to pursue at least one new avenue of science outreach in the coming year to promote transparency, share successes and contribute to a more informed public.**

- Get to know the communications officers at your organization or institution and their policies on press releases, and proactively reach out to print, online and radio media outlets.
- Take part in social media (e.g. Twitter, Instagram, Facebook, blogs) to highlight your own research and that of others.
- Participate in a contest that aims to highlight research progress and innovation (e.g. a research photography or video contest organized by a funding source).
- Inquire about preparing articles for an outreach section of a journal (e.g. *Conservation Physiology*’s upcoming ‘Conservation Physiology in Action’; *Journal of Experimental Biology*’s ‘Outside JEB’).
- Take time when preparing lay summaries or video abstracts, and petition journals you publish with to include them as a complement to their standard abstract requirements.
- If you organize a symposium for a conference, make public promotion part of your focus. For example, decide on an official hashtag for the symposium and publish it with the programme or online so that those who are media-savvy can easily use it.
- Attend a science communication workshop at a conference. If one is not offered, indicate to conference organizers your interest in having this type of workshop at future meetings.
- If you are a graduate student or early career researcher, find a mentor who has dedicated time to science communication and take advantage of their advice and tutelage regarding how to become more involved.
- Give a public presentation or write a newsletter article for a local community group (e.g. naturalists clubs, land trusts, non-government organizations, children's groups).
- Volunteer to teach the public about your research or the techniques you use (e.g. research showcases, Earth Day events, field laboratories, local hikes).
- Make or update your website and consider how it will be viewed by diverse audiences, rather than just colleagues.
- Take photographs of your field and laboratory work to compile an archive that you draw on when promoting your research.
- Contribute to or initiate a citizen science initiative.
- Take time to read science communication articles, gauge what is effective and incorporate these principles into your own writing and outreach.
- Push for the inclusion of public communication skills as part of the undergraduate science curriculum at your institution.
- Contact a policy-maker or sign a petition relating to a conservation issue that you are passionate about.

#### Keeping the explicit goal of contributing to policy change at the forefront

When the definition of conservation physiology was recently refined (Cooke *et al*., 2013), it explicitly stated that accomplishing ‘conservation’ constitutes ‘the development and refinement of strategies to rebuild populations, restore ecosystems, inform conservation policy, generate decision-support tools, and manage natural resources’. As a result, policy and management are integral pillars of conservation success for the field, and we suggest that researchers in the discipline constantly consider and articulate how their work can benefit a greater conservation goal.

#### Possessing an integrative framework that encourages inter-disciplinary research and collaboration

Linkage between multiple parties and decision-makers supports knowledge transfer, promotes the conceptualization of new ideas and enhances appreciation for different viewpoints. As a collaborative discipline by definition, conservation physiology can leverage these scenarios to translate their findings better, promote change and find the best ways to contribute to success. In particular, the field is increasingly emphasizing solution-oriented research questions that are co-created by scientists and societal stakeholders to promote new relationships and support a culture where science concurrently informs and learns from practice (Bai *et al*., 2016).

#### Highlighting its evidence base

As outlined above, proposals that tackle problems with methodologies that are recognized as successful can be the most convincing to funders, the general public and those who generate the policies needed to produce on-the-ground change (Sutherland *et al*., 2004). In particular, by recording and sharing our successes (as well as failures), it is possible to benefit evidence-based conservation and community engagement and to improve funding rates from public and private sources in order to solidify future conservation programmes.

#### Inspiring leaders who can connect with the public

As conservation physiology becomes a more established field, there will be a growing number of new faculty and other professionals who self-identify specifically with the discipline, who are also abreast of technological and social advances in communication. Conservation physiology inherently draws on cutting-edge technologies to monitor and manage the impacts of global change, providing engaging and hopeful stories to the public. We encourage both new and established conservation physiologists to connect with diverse audiences through online venues, print media and outreach activities (Box 2) as a way to share their research and passion for conservation. In particular, current leaders in the field can inspire a new generation of conservation physiologists to make public outreach a part of their normal workplace routine. This has the power to produce not only successful scientists, but also dynamic advocates who can generate an informed and engaged electorate, who will feel empowered to contribute to policy change. Members of the public can also be engaged in conservation physiology projects via citizen science (Dwyer *et al*., 2016), thereby generating opportunities to make meaningful and lasting changes in human behaviour.

## Conclusion

As conservationists, it is not surprising that the term ‘Anthropocene’ evokes negative connotations because we envision the damage that human population growth, landscape alteration and climate change have had on the natural systems and biodiversity that we aspire to preserve. However, as the field of conservation physiology continues to develop, participants have the power to balance warnings related to the plight of biodiversity with guidance for a positive, pragmatic, enlightened path forward. We are particularly hopeful, given the growing number of papers that move beyond the science of our discipline to highlight the boundary between science and action (see Cooke *et al*., 2013; Nguyen *et al*., 2016). Physiological research is increasingly being conducted in partnership with practitioners, policy-makers and stakeholders who are required for relevance, knowledge mobilization and meaningful change. By envisaging how to define and achieve the best Anthropocene possible, we hope that other researchers and practitioners will be motivated, optimistic and increasingly productive as they continue to apply physiological techniques to achieve diverse conservation goals.
